# Coordinated leaf anatomy buffers the effects of reduced stomatal density on CO_2_ diffusion

**DOI:** 10.1093/plphys/kiag434

**Published:** 2026-06-25

**Authors:** Yuzhen Fan

**Affiliations:** Assistant Features Editor, Plant Physiology, American Society of Plant Biologists; Division of Plant Sciences, Research School of Biology, Australian National University, Canberra, ACT 2601, Australia

Stomata are microscopic pores on the leaf surface that regulate the exchange of carbon dioxide (CO_2_) and water vapor (H_2_O) between leaves and the atmosphere. They are the gatekeepers of leaves and play a crucial role in supplying CO_2_ to the site of photosynthesis. However, this comes at the cost of H_2_O loss through transpiration because the atmospheric gradient driving H_2_O diffusion is much steeper than that for CO_2_ (Cowan and Farquhar 1977). This tradeoff becomes particularly acute under hot and dry conditions, where high evaporation increases transpirational H_2_O loss and leads to stomatal closure, thereby reducing CO_2_ gain and photosynthesis. Consequently, considerable effort has been made to improve leaf-scale water-use efficiency (WUE) by reducing transpirational H_2_O loss while maintaining CO_2_ assimilation. For example, a decrease in stomatal density or an increase in the speed of stomatal opening or closure could improve WUE and drought tolerance (Lawson and Blatt 2014). However, changes in stomatal traits could also alter internal leaf anatomy, potentially affecting CO_2_ diffusion within the leaf and thus affecting photosynthesis.

After entering the leaf through stomata, CO_2_ then diffuses through an irregular network of internal airspaces to reach the site of photosynthesis in mesophyll cells. The efficiency of this process (ie internal airspace conductance to CO_2_; *g*_ias_) is influenced by a number of factors, including the chance that CO_2_ enters through stomata instead of epidermis, how CO_2_ moves through airspace from the entry point of a stomate, and the volume ratio of airspace to mesophyll cells. In general, greater stomatal density leads to a larger volume of substomatal crypts (ie the bulk of porous space within the leaf functions as a diffusive pathway for CO_2_; see Fig. 1), a higher volume ratio of airspace to mesophyll cells, and faster *g*_ias_ (Lundgren et al. 2019; de Oliveira et al. 2022). The placement of stomata on the leaf surface also matters to internal CO_2_ diffusion. Most plants distribute stomata exclusively to the lower surface of the leaf, whereas a small number of species possess stomata on both leaf surfaces (Harrison et al. 2020). In addition, stomata generally avoid developing above vascular bundles because the limited space between the epidermis and the underlying bundle sheath would restrict the formation of substomatal crypts (Schuler et al. 2018). Together, it is suggested that there is a close relationship among stomatal density, the placement of stomata, and internal leaf anatomy. *g*_ias_ is expected to differ between the upper and lower leaf surfaces with distinct stomatal density.

**Figure 1:**
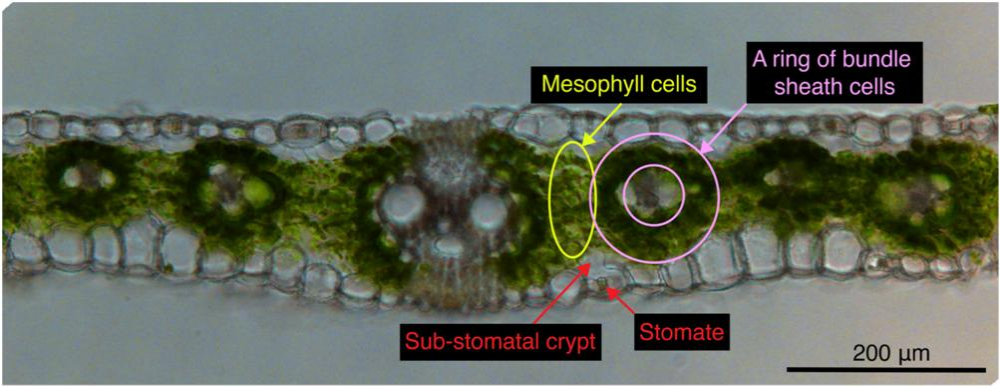
Bright-field light micrograph of a leaf cross-section from sorghum. Leaf tissue was hand-sectioned and imaged using a Leica SP8 confocal microscope by Y. Fan.

A major limitation to studying the relationship between external traits (eg stomata) and internal anatomy (eg airspace) of a leaf has been the capacity for visualizing the leaf interior in 3 dimensions (3D) at sufficiently high resolution. Recently, in *Plant Physiology*, Fischer et al. (2026) combined high-resolution X-ray micro-computed tomography (microCT) with machine learning to characterize mesophyll, epidermis, and airspace anatomy, revealing how stomatal patterning is coordinated with 3D leaf structure. They also explore how variation in stomatal density influences internal leaf anatomy and *g*_ias_ by comparing a transgenic *Sorghum bicolor* (sorghum) line with 45% to 69% less overall stomata (Ferguson et al. 2024) with its wild-type counterparts.

Their findings challenge hypotheses that were formed based on the literature. First of all, Fischer et al. (2026) discovered that stomata on the upper surface of the leaves are distributed over rather than between vascular bundles. This result is surprising, though it is possible that rows of bulliform epidermal cells occupy the interveinal regions of the upper-leaf epidermis, potentially pushing stomata toward positions overlying vascular bundles. Second, stomata over vascular bundles were associated with markedly smaller substomatal crypts on the upper leaf surface, resulting in the corresponding *g*_ias_ values that were substantially lower than those predicted from stomatal density alone. Third, transgenic sorghum lines with reduced stomatal density developed larger stomata and substomatal crypts, yet maintained *g*_ias_ at levels comparable with the wild type. This is particularly true for the lower leaf surface, where the crypts were voluminous, and *g*_ias_ was well-maintained even when lower-surface stomatal density was reduced. Overall, these results highlight that a potential compensation mechanism exists whereby changes in stomatal and crypt anatomy offset the potential negative effects of reduced stomatal density on *g*_ias_. It is possible that the larger stomata and crypts may have compensated for the increased diffusion distances associated with reduced stomatal density. As a result, *g*_ias_ remained unchanged, revealing an unexpected degree of anatomical plasticity in sorghum leaves.

Findings of Fischer et al. (2026) have a substantial impact on stomatal engineering in the context of improving crop WUE and productivity. As mentioned above, reducing stomatal density has emerged as a promising target for decreasing transpirational H_2_O loss. However, the concerns have always been that reductions in stomatal density could limit CO_2_ uptake and affect CO_2_ diffusion within the leaf. Fischer et al. (2026) presents evidence that reductions in stomatal density do not necessarily alter CO_2_ diffusion within the leaf due to coupled changes in stomatal patterning, crypt anatomy, and the 3D organization of internal airspace, mitigating the effect of stomatal density on *g*_ias_. This suggests that alteration of stomatal density may trigger coordinated developmental responses throughout the leaf, highlighting the need for more comprehensive analyses of internal leaf anatomy when exploring stomatal engineering strategies. Visualization technologies, such as high-resolution microCT, serial block-face scanning electron microscopy, and confocal microscopy (Khoshravesh et al. 2022), when combined with advances in computational analysis, provide a powerful suite of tools for revealing the structure-function relationships that underpin leaf physiology.

**Recent related articles in *Plant Physiology*:**

- Crawford et al. (2025) used Stomata In-Sight, a system for simultaneous measurement of stomatal aperture and leaf gas exchange, and showed that variation in stomatal conductance could be largely explained by pore apertures in maize.
- Zailaa et al. (2024) investigated how minimum leaf conductance responded to elevated temperature and vapor pressure deficit in 24 *Quercus* species. They found that minimum leaf conductance declined in dry, hot conditions, largely due to stomata closing more tightly.

## Data Availability

No new data were generated or analysed in support of this News and Views article.
